# Persistent dysphagia is a rare problem after laparoscopic Nissen fundoplication

**DOI:** 10.1007/s00464-018-6396-5

**Published:** 2018-08-31

**Authors:** Milena Nikolic, Katrin Schwameis, Georg Semmler, Reza Asari, Lorenz Semmler, Ariane Steindl, Berta O. Mosleh, Sebastian F. Schoppmann

**Affiliations:** Division of General Surgery, Department of Surgery, Upper-GI-Research and Service, CCC-GET, Medical University of Vienna, Waehringer Guertel 18-20, 1090 Vienna, Austria

**Keywords:** Dysphagia, Gas-bloat syndrome, Heartburn, Fundoplication, Gastroesophageal reflux disease

## Abstract

**Background:**

Although around 30% of patients with gastroesophageal reflux disease (GERD) are insufficiently treated with medical therapy, only 1% opt for surgical therapy. One of the reasons behind this multifactorial phenomenon is the described adverse effect of long-term dysphagia or gastric bloating syndrome after surgical treatment. Aim of this study was to evaluate the most common side effects associated with anti-reflux surgery, as well as long-term outcomes in a large cohort of highly surgically standardized patients after laparoscopic Nissen fundoplication (LNF).

**Methods:**

Out of a prospective patients’ database including all patients that underwent anti-reflux surgery between 01/2003 and 01/2017 at our institution, 350 consecutive patients after highly standardized LNF were included in this study. A standardized interview was performed by one physician assessing postoperative gastrointestinal symptoms, proton pump inhibitor intake (PPI), GERD-Health-Related-Quality-of-Life (GERD-HRQL), Alimentary Satisfaction (AS), and patients’ overall satisfaction.

**Results:**

After a median follow-up of 4 years, persistent dysphagia (PD) after LNF was observed in 8 (2%) patients, while postoperative gas-bloat syndrome in 45 (12.7%) cases. Endoscopic dilatation was needed in 7 (2%) patients due to dysphagia, and 19 (5%) patients underwent revision surgery due to recurrence of GERD. The postoperative GERD-HRQL total score was significantly reduced (2 (IQR 0–4.3) vs. 19 (IQR 17–32); *p* < 0.000) and the median AS was 9/10. Heartburn relief was achieved in 83% of patients. Eighty-three percent of patients were free of PPI intake after follow-up, whereas 13% and 4% of the patients reported daily and irregular PPI use, respectively.

**Conclusion:**

LNF is a safe and effective surgical procedure with low postoperative morbidity rates and efficient GERD-related symptom relief. PD does not represent a relevant clinical issue when LNF is performed in a surgical standardized way. These results should be the benchmark to which long-term outcomes of new surgical anti-reflux procedures are compared.

Gastroesophageal reflux disease (GERD) is one of the most common gastrointestinal disorders, reaching a prevalence up to 30% in the Western World [1–8]. Although the gold standard in treatment remains surgical repair by laparoscopic fundoplication, patients opt to undergo surgery only when first-line treatment with proton pump inhibitors (PPIs) fails and quality of life is reduced or a risk of developing adenocarcinoma develops [4, 5, 8, 9]. One of the reasons behind a low surgery rate among GERD patients is the fear of its adverse effects [3, 4, 9]. Temporary side effects due to transient postoperative mucosal edema include dysphagia and increased bloating [7–11]. However, three to 24% of patients who underwent laparoscopic fundoplication continue to report persistent dysphagia (PD), making it one of the leading causes of procedure failure [7, 12, 13]. While the precise etiology of PD remains unknown, possible reasons are believed to be a tight hiatus or slipped fundoplication, as well as preoperative esophageal motility disorders [8, 9, 13, 14].

In an effort to reduce PD rates while maintaining effective reflux control alterations of the standard Nissen fundoplication have been developed over time [10]. Such modifications involve partial 270° Toupet fundoplication (TF) or anterior 120° Dor fundoplication (DF) [10, 15]. TF and DF have both been associated with lower PD rates, while only TF has shown similar efficacy to LNF in reflux control [8, 13, 15]. Nonetheless, no clear consensus exists if and when TF should be selected over a LNF and if the described postoperative reduction in dysphagia rates remains in the long term. Thus, far LNF remains superior to TF and DF in long-term reflux cessation and symptom relief [15–17].

Besides alterations of the standard fundoplication, innovative, less invasive surgical treatment options, such as magnetic sphincter augmentation (MSA) and lower esophageal sphincter stimulation (LESS), are being utilized more each day. Currently, in a commencing time of individualized anti-reflux surgery, it is essential to reevaluate long-term results of the standard therapy and create a new touchstone for future research.

Aim of this study was to analyze PD rates, reflux control, and degree of overall satisfaction in GERD patients who underwent highly standardized LNF in a high input specialized reflux center.

## Methods

All 795 GERD patients collected in a prospectively created database that had undergone anti-reflux surgery between 01/2003 and 1/2017 at our institution were identified. Patients with hemi-fundoplication, endoscopic anti-reflux plastic surgery, magnetic or electric sphincter augmentation were excluded. Finally, all patients that underwent LNF (*n* = 350) were included into this study.

This study was approved by the Institutional Review Board of the Medical University of Vienna, Austria.

### Preoperative assessment

Preoperative, all patients had received a standardized interview, clinical examination, an upper GI endoscopy, a video esophagram, and esophageal functioning testing consistent of a manometry and a 24-h Impedance–pH-metry. GERD was defined by positive pH results or positive symptom correlation on esophageal functioning tests, the presence of esophagitis on endoscopy or typical GERD symptoms sensitive to PPI medication.

### Surgery

All procedures were performed by four surgeons part of a specialized upper gastrointestinal team. The surgical approach was laparoscopic in all cases.

LNF was performed in a highly standardized technique as described shortly: Five trocars were placed in the upper abdominal wall in a standardized manner regarding surgeon’s and patient’s positions (anti-Trendelenburg), further trocar sites, and used instruments. A single instrument was used to retract the left lobe of the liver and expose the esophageal hiatus.

Both crus of the diaphragm were dissected using the Ultrasonic dissector in order to expose the distal esophagus. Special care was taken to achieve an adequate “intraabdominalisation” of the lower esophagus of at least 3 cm in length. A posterior hiatal closure without esophageal compression was performed with 2–5 stitches using non-absorbable single knot sutures without bougie in the esophagus. An extra-short warp, measuring 1.5 cm in a maximum with the naked eye was created using 2 close stitches with non-absorbable sutures. Division of the small gastric vessels was not performed when the construction of a tension-free wrap was possible and special care was taken to complete mobilization of the fundal adhesions to the diaphragm. The first stitch included the anterior esophageal wall. The vagal nerve was always identified and included in the wrap. In 80% of patients, a right posterior fundophrenicopexia was performed. After the surgery was completed, a blunt laparoscopic instrument was placed through the posterior esophageal wall and the wrap in order to determine the looseness of the fundoplication.

Postoperative, all patients received a restricted semiliquid food diet for the first 10 days, slowly progressing to solid food in order to avoid dysphagia during the development of mucosal edema. After at least one overnight stay, patients were discharged from the hospital once they were showing an unremarkable postoperative barium swallow.

### Postoperative assessment

**Fig. 1:**
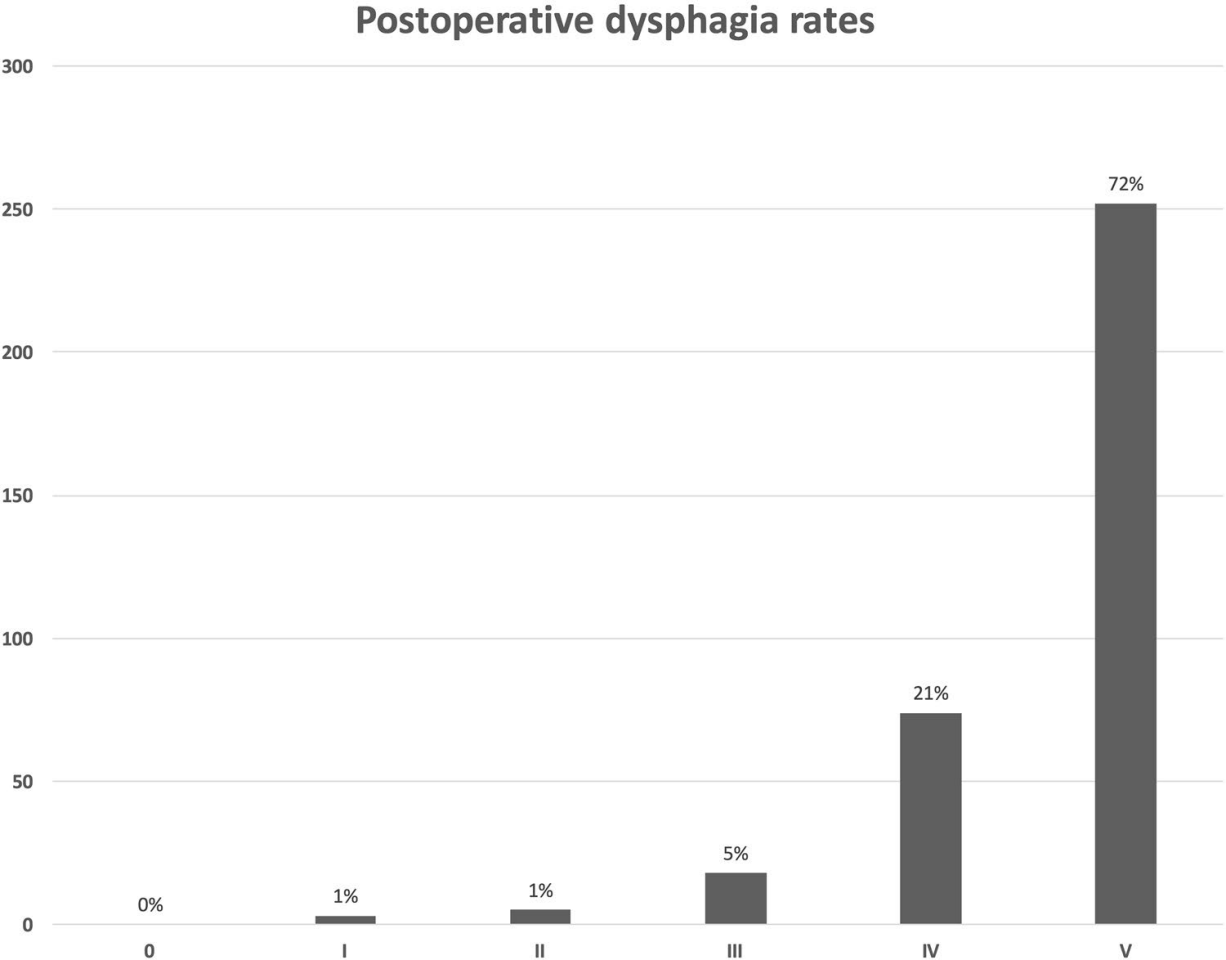
Frequency and degree of postoperative dysphagia based on the classification of Saeed et al. Columns from left to right: 0 = Unable to swallow (0); I = Swallowing liquids with difficulty, solids impossible (3); II = Swallowing liquids without difficulty, solids impossible (5); III = Occasionally difficulty swallowing with solids (18); IV = Rarely difficulty swallowing with solids (74); and V = Swallowing normally (252)

On the first postoperative day, a barium swallow was performed in all patients. Follow-up was performed by the same physician using a standardized interview assessing postoperative gastrointestinal symptoms, proton pump inhibitor intake (PPI), GERD-Health-Related-Quality-of-Life (GERD-HRQL), Alimentary Satisfaction (AS), and patients’ opinions weather they were willing to undergo the same procedure in same circumstances. The frequency and severity of postoperative dysphagia was assessed using the classification of Saeed et al., where the ability to swallow can be scored from 0 to 5, where 0 is inability to swallow and 5 is normal swallowing (Fig. 1) [18].

Adverse effects such as complications, hospital readmission, emergency surgery, or elective re-operation were documented. Patients with recurrent symptoms received upper GI endoscopy as well as esophageal functioning tests.

### Statistical analysis

Statistical analysis was performed using SPSS® statistics 20.0 (IBM, Armonk, NY). Data were described using median (interquartile range) or mean (range). Statistical analysis appropriate for non-parametric data was used. Categorical variables were assessed using the Fisher exact test and continuous data using the Wilcoxon Rank test as appropriate. Statistical significance was defined as a *p* value < 0.05.

## Results

A total of seven hundred and nighty-five patients underwent anti-reflux surgery (*n* = 795) for chronic gastroesophageal reflux disease in a period of 14 years in our specialized upper gastrointestinal surgery center. Five hundred and nighty-eight of them underwent modified LNF. At time of follow-up, sixty-two patients (*n* = 62) were not reachable, sixteen (*n* = 16) deceased, and thirteen (*n* = 13) refused to take part in the follow-up. Ultimately, a total number of three hundred and fifty (*n* = 350, 193 male and 157 female) patients were left in our study. The median age was 53 years (IQR 41–62) and the median preoperative BMI was 27 (IQR 24–29). A hiatal hernia was present in two hundred and ninety (*n* = 290, 83%) of the patients, which of thirty-five (*n* = 35, 12%) were paraesophageal hernias. Fourteen (*n* = 14, 40%) of the paraesophageal hernias were noted as Upside-down stomach, a type III hernia using the classification of Hill et al. [19].

All patients had received preoperative esophageal functioning tests (EFTs). An anti-reflux surgery was indicated when the total pH percentage time was increased (pH > 4.2%) and/or there was increased total reflux episodes (> 73) with positive symptom correlation. The median total pH percentage time was 7.6% (IQR 4.1–15.1%), while the median total reflux episodes was 65 (IQR 43–94). According to the Chicago Classification v3.0 ineffective esophageal motility (IEM) was seen in sixty-three (*n* = 62, 18%) of the patients prior to surgery. We found no difference in the postoperative PD rate in patients diagnosed with IEM preoperatively and those with intact motility (*n* = 2, 3% vs. *n* = 6, 2%, χ^2^ = 0.298, *p* = 0.585).

In the preoperative histology, taken upon esophagogastroduodenoscopy (EGD), fifty-two (*n* = 52, 15%) patients showed Barrett’s metaplasia.

The three most common GERD-associated preoperative symptoms were heartburn (*n* = 299, 85%), regurgitations (*n* = 205, 59%), and dysphagia (*n* = 52, 15%). A total of three hundred and thirty-three (*n* = 333, 95%) of the patients reported the use of PPIs prior to surgery. Demographics and preoperative findings are shown in Table 1.

**Table 1:** Demographic data and results of preoperative diagnostics

	Total *n* = 350 (100%)
Sex (m vs. f)	193 (55) vs. 157 (45)
Median age (IQR)	53 (41–62)
Presence of hiatal hernia	290 (83)
Paraesophageal hernia	35 (12)
Upside-down stomach	14 (40)
Median BMI (IQR)	27 (24–29)
EFT performed in our center	338 (97)
Median pH percentage time (IQR)	7.6 (4.2–15.1)
Median total reflux episodes (IQR)	65 (43–94)
IEM	62 (18)
Barrett’s esophagus	52 (15)
Preoperative PPI use	333 (95)
Median GERD-HRQL total score	19 (12–25)
Symptoms	
Heartburn	299 (85)
Regurgitations	205 (59)
Dysphagia	52 (15)

The median operation time (OR) time was 85 min (range 30–275). The surgical approach was laparoscopic in all patients. No intraoperative complications were seen. All patients received hiatoplasty, whereas in forty-five patients (*n* = 45, 13%) a hiatal mesh was implanted.

**Fig. 2:**
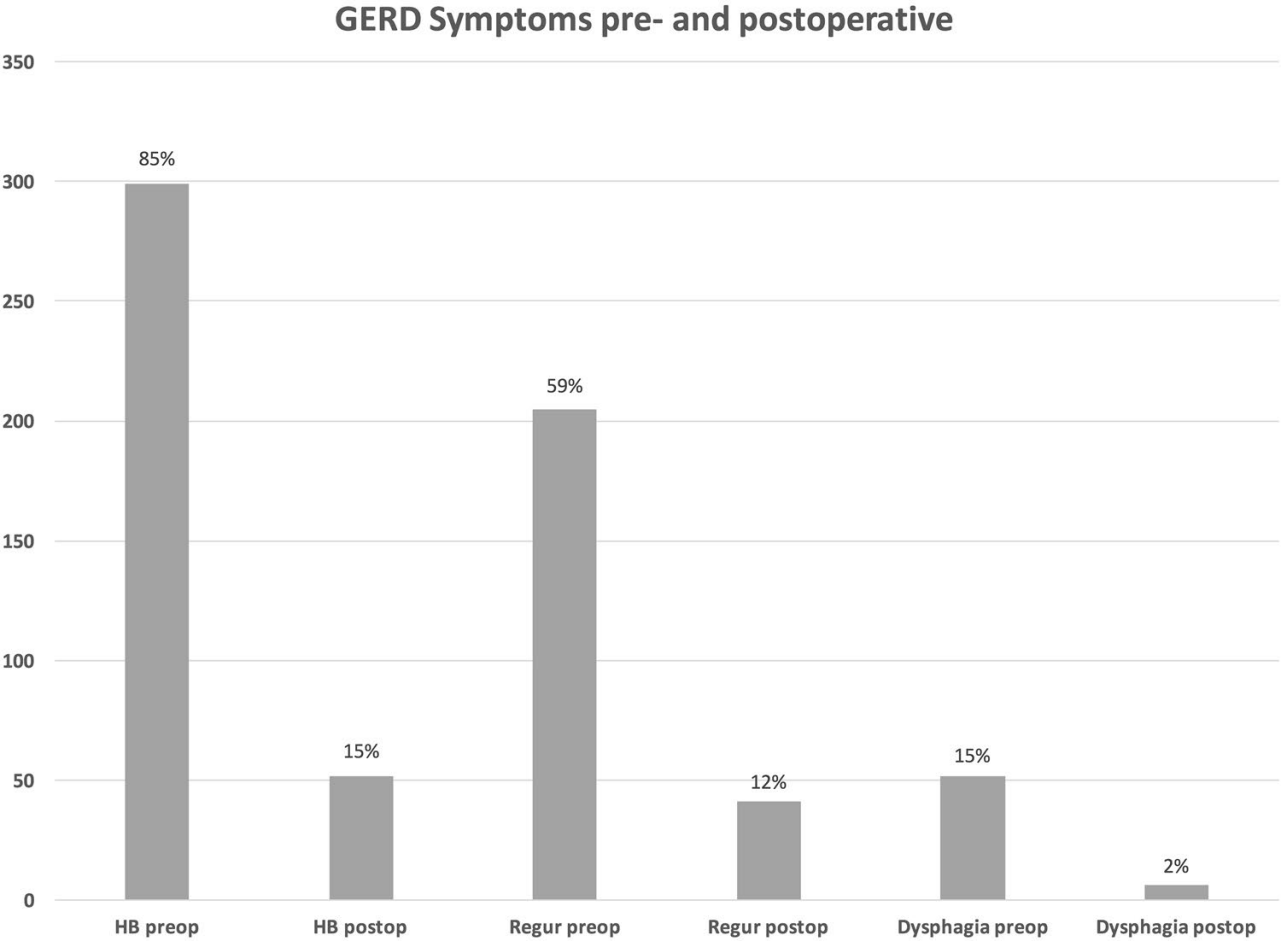
Comparison of most common pre- and postoperative symptoms

The median follow-up time was 4 years (IQR 2–8). Heartburn, regurgitations, and dysphagia were fully eliminated in two hundred and seven (*n* = 247, 84%), one hundred and sixty-four (*n* = 164, 80%), and forty-six (*n* = 46, 88%) of the patients, respectively (Table 2). A graphic comparison of the three most reported symptoms before and after modified LNF is shown in Fig. 2.

**Table 2:** Postoperative symptom relief

Total *n* = 350 (100%)	Symptom relief: *n* (%)
Heartburn (HB)	247/299 (83)
Regurgitations	164/205 (80)
Difficulty swallowing	46/52 (88)

**Fig. 3:**
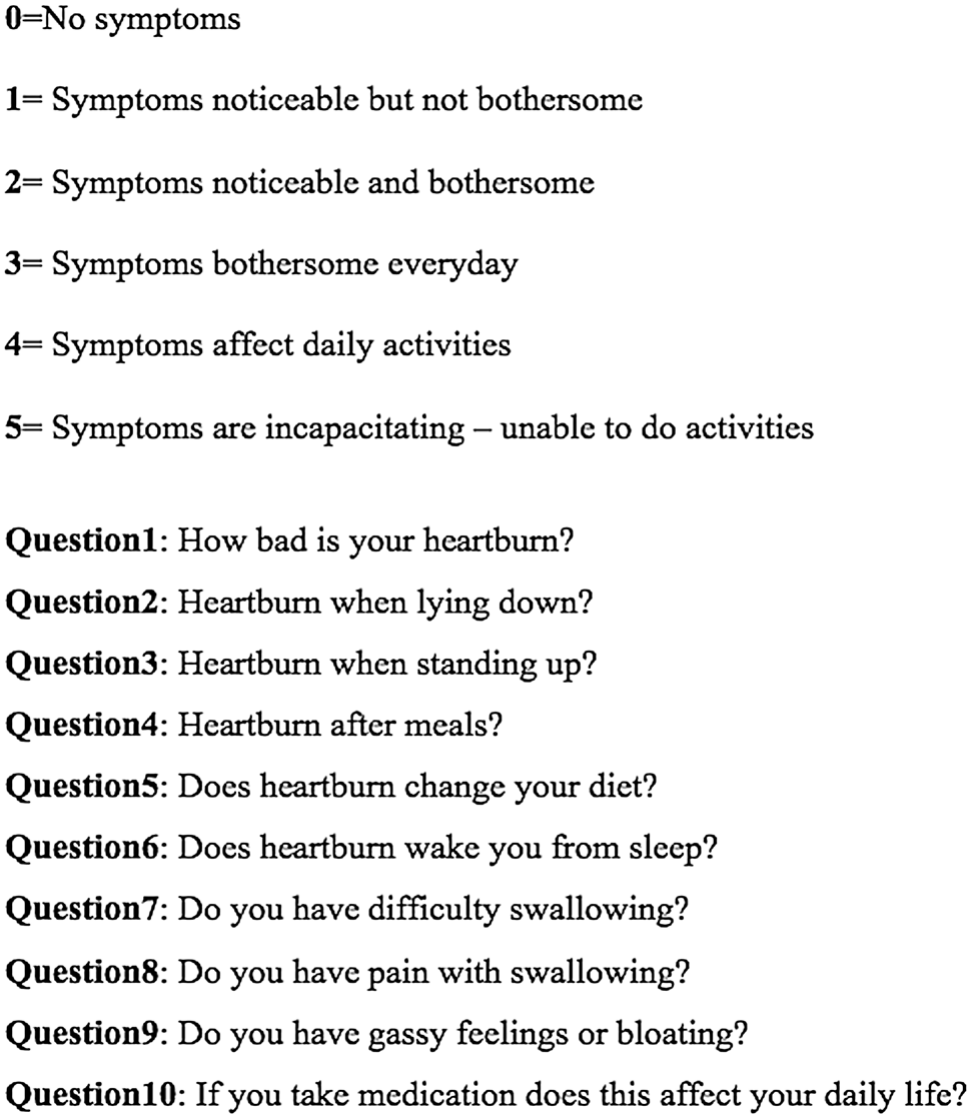
GERD-HRQL questionnaire

**Fig. 4:**
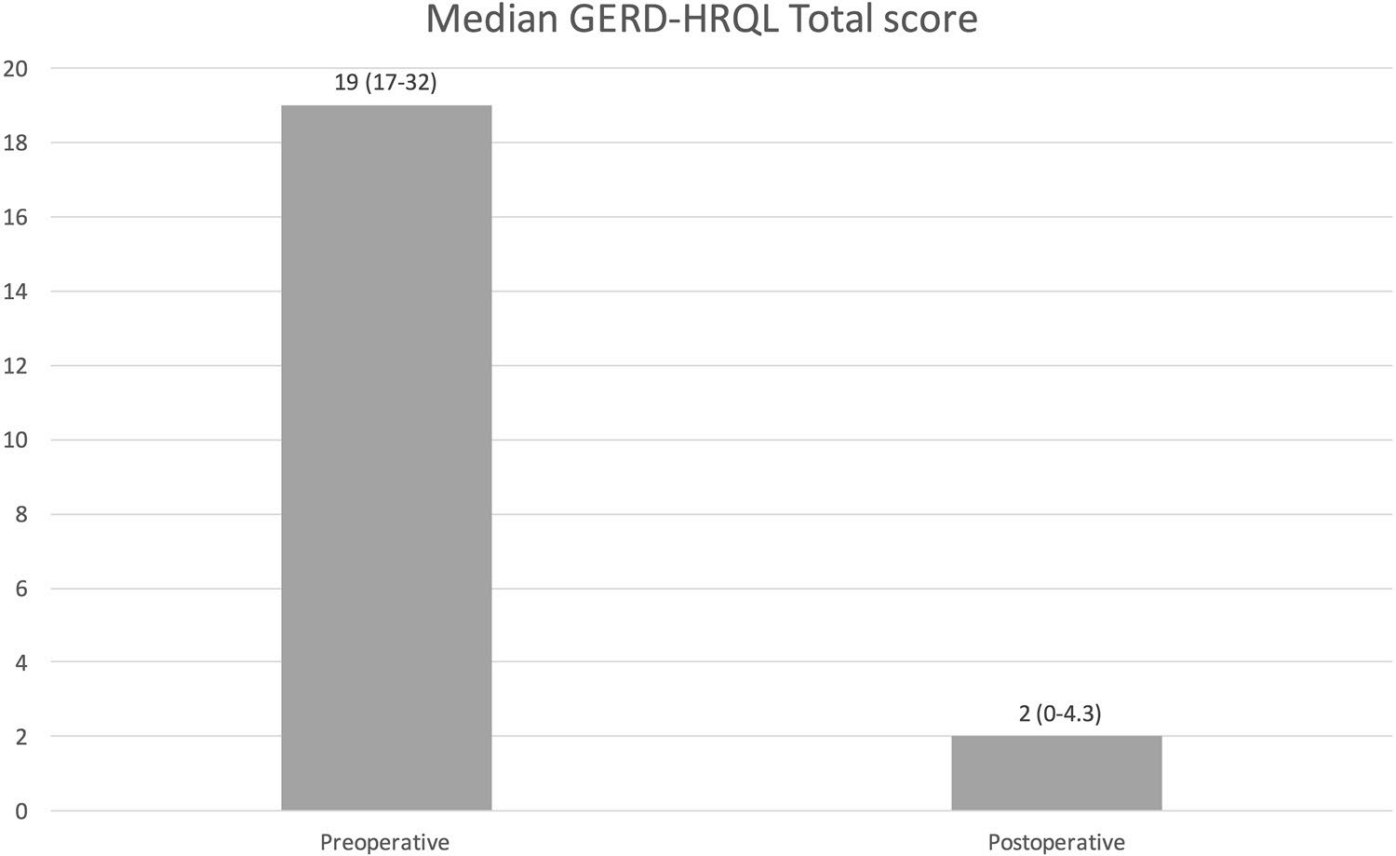
Comparison of pre- and postoperative median GERD-HRQL total score. The lower the score is, the higher the quality of life is

Prior to surgery, 92 patients had completed the GERD-HRQL score (Fig. 3). The preoperative median GERD-HRQL was 19 (IQR 12–25). LNF led to a significant reduction of the GERD-HRQL total score (2 (IQR 0–4.3) vs. 19 (IQR 17–32); *p* < 0.000). (Fig. 4). Moreover, the median alimentary satisfaction (AS) was rated 9. This proves a substantial increase in quality of life in our patients. Satisfaction with heartburn relief was achieved in two hundred and sixty-six (*n* = 266, 95%) of the cases. Two hundred and eighty-nine (*n* = 289, 83%) patients reported to be completely free of PPIs postoperatively, while fourteen (*n* = 14, 4%) patients reported occasional PPI intake and forty-seven (*n* = 47, 13%) patients needed regular PPI use. When asked if they would be willing to undergo the same surgery, in same circumstances, two hundred and seventy-nine (*n* = 279, 79%) patients said yes. Postoperative outcomes and Quality of Life results are presented in Table 3.

**Table 3:** Postoperative outcomes and quality of life results

Median GERD-HRQL total score	2 (IQR 1–4.7)
Median alimentary satisfaction (AS)	9/10
Satisfaction with heartburn relief	283/299 (95)
Postop. outcome rated excellent/good	293 (83)
Postoperative PPI relief	273 (82)
Postoperative BMI	26 (24–28)
Would undergo surgery again	278 (79)

Persistent dysphagia (PD) was reported in eight (*n* = 8, 2%) patients. Rarely difficulties swallowing with solids only were reported by seventy-three (*n* = 73, 21%) of the patients, while twenty (*n* = 20, 6%) patients had occasional difficulties swallowing with solids. The frequency and severity of postoperative dysphagia based on the classification of Saeed et al. is shown in Figure 1. A whole of two hundred and fifty-seven (*n* = 257, 73%) of the patients retained their ability to belch/vomit and forty-five (*n* = 45, 13%) complained about increased daily gas bloating.

PD was described in two patients where postoperative diagnostics (barium swallow, real time MRI, and EGD) showed no morphologic abnormalities so that no re-operation was indicated, but endoscopic dilatation and Botox injection, respectively. One patient experienced recurrence of symptoms 1 year postoperatively. A real-time swallow MRI revealed a disrupted wrap. This was an indication for a re-fundoplication, after which the patient developed dysphagia. A subsequent barium swallow showed delay contrast passage so that a third revision was indicated. Intraoperatively no stenosis, slipping, or disposition of the wrap was seen, so that after adhesiolyses the procedure was terminated. One patient developed a postoperative stenosis in the cardia and after multiple ineffective endoscopic dilatations, a cardiomyotomy and conversion to Toupet fundoplication was ultimately performed.

**Fig. 5:**
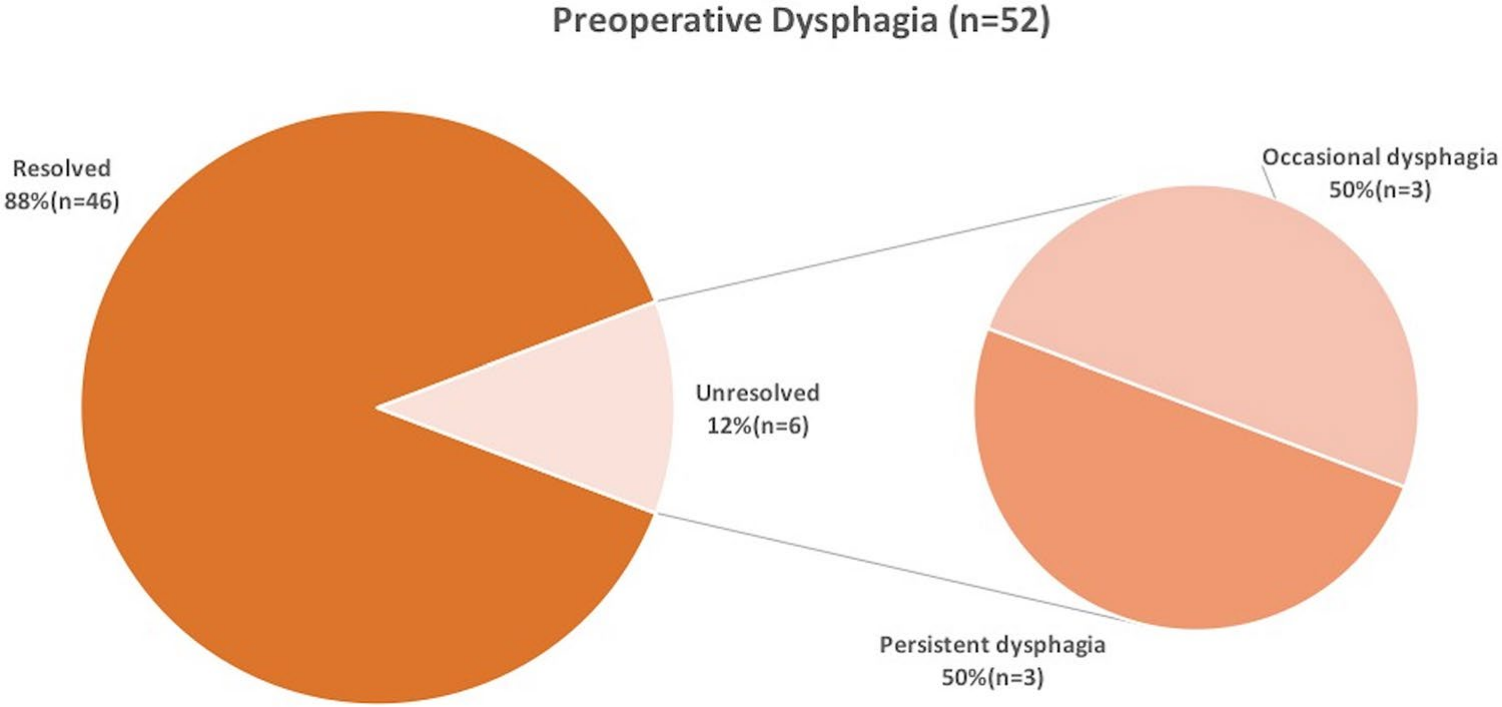
Frequencies of resolved and unresolved preoperative Dysphagia

**Fig. 6:**
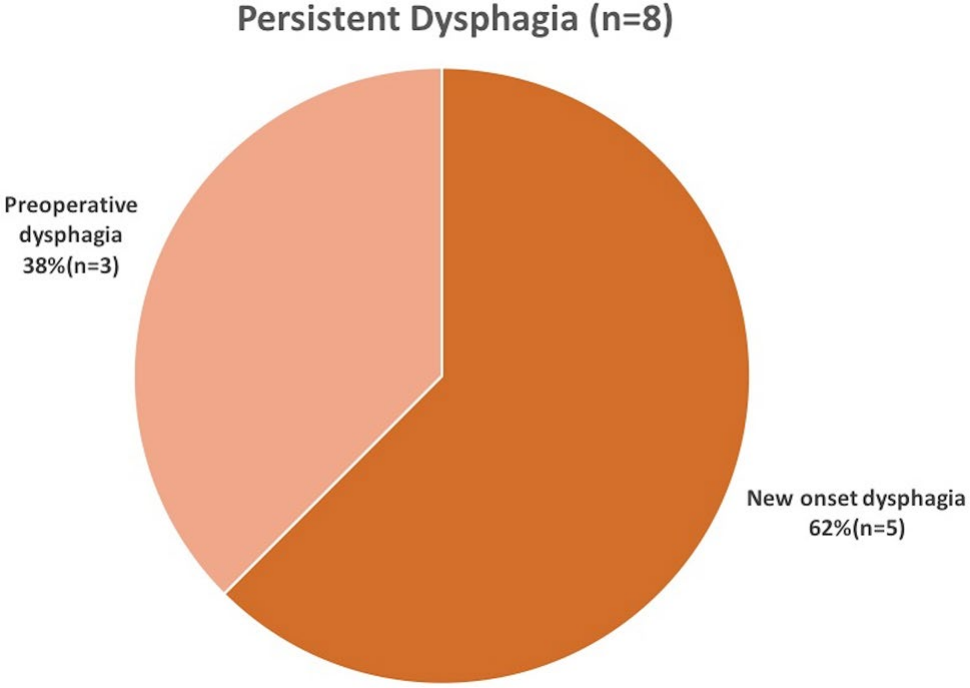
Distribution of Persistent dysphagia between new onset dysphagia and preoperative dysphagia

Further two cases of PD were due to slipping of the wrap. One confirmed on VEG, MRI, and EGD, but due to the patient’s desire for no re-operation, no surgical measures were taken, while the other underwent revision surgery in a different institution. Lastly, two patients, without any preoperative risk factors or anatomical correlation developed PD and desired no further evaluation. The frequencies of resolved and unresolved preoperative dysphagia as well as the distribution of PD between new onset and preoperative dysphagia are shown in Figs. 5 and 6.

Endoscopic dilatation was successfully performed in five patients with postoperative dysphagia (*n* = 5/7, 71%). Nineteen (*n* = 19, 5%) patients required re-fundoplication surgery. Fourteen due to recurrence of reflux and the hiatal hernia, two due to slipping of the fundoplication, one due to rupture of the wrap, one due to an acute paraesophageal hernia recurrence on day 2 postoperatively, and lastly one conversion to Toupet Fundoplication due to PD was observed.

## Discussion

The only definite treatment of GERD and prevention of its severe long-term consequences is surgical enhancement and reconstruction of the weakened LES, inhibiting reflux of gastric contents in to the esophagus. So far, the LNF has been the standard anti-reflux surgery with proven long-term efficacy and safety as well as verified improvement in quality of life in patients undergone LNF [4, 5, 8, 9, 20]. Nevertheless, it is associated with several complications like PD and gas-bloat syndrome, potentially leading to further surgical interventions and patient dissatisfaction [3, 7–11, 20]. In an effort to decrease the gap between medical and surgical GERD therapy by reducing the risk of potential side effects, magnetic sphincter augmentation (MSA) as well as lower esophageal sphincter stimulation (LESS) have come to use. As further treatments against GERD develop, it is crucial not to underestimate the golden standard and have valid, up-to-date long-term outcome results, that will serve as the benchmark for further research [3, 21–24].

Postoperative dysphagia may present in several ways: acute dysphagia due to slipping or rupture of the wrap, mild dysphagia 6–8 weeks after surgery due to local edema and/or hematoma, and persistent dysphagia occurring beyond 8 weeks, of which pathogenesis is still unknown. Mild dysphagia is relatively usual in the direct postoperative phase and improves as the mucosal edema and/or hematoma of the wrap subside. Instead, PD is infrequent, but occurs up to 20% and represents a challenge in further treatment [4, 9, 10]. It has been shown that the presence of preoperative dysphagia and IEM, certain preoperative LES characteristics and surgical technique have an influence in the development of PD [9, 10, 25].

Although there is no clear consensus on the ideal fundoplication, lower PD rates have been observed in patients who underwent TF instead of LNF [8, 10, 15]. Furthermore, TF is the current treatment of choice in patients with IEM, in order to avoid PD [8]. However, LNF still remains to be the most effective anti-reflux operation with best GERD-symptom control and reflux elimination rates [15–17]. Studies have shown that not only the type of fundoplication, but also the length of the wrap effects the prevalence of PD. Thus, the policy of a thin Nissen sleeve, measuring not longer than 1.5–2 cm has been established [10, 26].

In order to prevent PD while achieving adequate symptom relief, at our center all patients underwent the same procedure: a 1.5–2 cm, 360°, tension free, wide wrap with modified technical details. Although including the division of the short gastric vessels while performing fundoplication is believed to facilitate in the construction of a tension-free wrap, thus minimizing the risk of PD [27], published data on this issue remain controversial. Various randomized controlled studies have shown no impact of dividing the short gastric vessels on postoperative dysphagia rates [28–33]. Moreover, increased length of surgery and a higher incidence of postoperative bloating have been described for patients that underwent division of the short gastric vessels on principle [34–36]. In our series, the short gastric vessels were left intact, where the construction of a tension-free wrap was possible.

The intraoperative use of a bougie while constructing the wrap was primarily encouraged by a study in 1986 showing an association between the use of a larger bougie and lower temporary postoperative dysphagia rates [37]. Even though these primarily data were confirmed in a small prospective randomized study [38], certain limitations of the study such as additional concurrent laparoscopic procedures that 34 patients underwent (unknown in which group), as well as the validity of the scoring system used to assess the dysphagia should be taken into consideration. Additionally, as various further studies reported no benefit of using the bougie in postoperative dysphagia rates and the potential benefit is opposed by the risk of esophageal perforation, the Society of American Gastrointestinal and Endoscopic Surgeons give a Grade B recommendation for the supplementary procedure [39–43]. Taken the moderate evidence for the use of the bougie, as well as associated risks into consideration, we chose against its use in our LNF procedure.

The first suture involved the anterior esophageal wall, decreasing the risk of migration or slipping. As the esophagus is fixated in its position, the anterior hiatoplasty is not needed, leaving space for the esophagus to expand during swallowing. Due to the fact that all surgeries were performed laparoscopically and surgical laparoscopic skills improved throughout the 14 years, a wide range in the OR time (range 30–275) exists. Likewise, being a teaching university hospital has its effect on the OR time. Other than one of the four highly specialized surgeons always being present during the operation, interns and residents took part as well.

In our study, we observed only eight (*n* = 8, 2%) patients with PD, which seems significantly lower than previously described in literature. Bonadiman et al. reported a PD rate of 3.11% in a retrospective study comparing anterior and posterior gastric wall fundoplication, finding no significant difference between the groups [11]. Richter et al. found 3–24% of patients developing PD after LNF [44]. In an 11-year follow-up by Schietroma et al., 4% developed severe dysphagia after LNF [20]. Lastly in a prospective European study comparing medical to surgical treatment of GERD, a PD rate of 11% was observed [45]. The definite cause of PD was found in two patients: slipping of the wrap in one case and peptic stenosis in the other. Further, two patients had no anatomical or structural correlate found as the possible cause of dysphagia. Three of the patients reported experiencing dysphagia preoperatively. The data have been controversial on whether or not subjective preoperative dysphagia increases the risk of postoperative PD [13]. Two of the patients were diagnosed preoperatively with IEM, which has been described as a risk factor for postoperative PD [8, 13]. Although previous studies have demonstrated lower PD rates after partial fundoplication, making it the preferred surgical approach in patients with IEM [8, 10, 15], available evidence suggests that the outcomes of patients with IEM are not affected by the type of fundoplication [46–49]. We confirm these findings as no significant difference was found in postoperative PD rates when compared between the two subgroups (*n* = 2, 3% vs. *n* = 6, 2%, *χ*^2^ = 0.298, *p* = 0.585). Nevertheless, further prospective randomized analyses are needed to confirm these findings.

In our series, only two patients (*n* = 2, 0.5%) underwent re-operation due to dysphagia, where by during one revision only adhesiolyses was performed as no intraoperative abnormalities could be found. A total of seven cases received endoscopic dilatation (*n* = 7, 1.4%). Both rates lower when compared to previous international studies. Laffularde et al. reported a revision rate of 3.9% and a 5% rate of endoscopic dilatation, while Schietrome et al. described re-operation in 1.6% and endoscopic dilatation in 2.2% of patients due to dysphagia [20, 50].

Another, incompletely understood complication of LNF is gas-bloat syndrome, usually accompanied by the inability to belch or vomit. Some possible explanations for its occurrence are thought to be intraoperative manipulation of the vagal nerves, a too tight fundoplication wrap and gastric emptying disorder. The incidence can vary, with a rate of up to 85% described in previous literature [8, 20]. We defined gas-bloat syndrome as bloating that was rated as “bothersome every day,” “effects daily activities,” or “incapacitating-unable to do activities” and observed a rate of 12.7%. A whole of 73% of our patients still preserved their ability to belch and vomit.

The main objectives in GERD treatment are symptom relief and mucosal healing. The most prevalent symptoms observed were heartburn, regurgitation, and dysphagia, coinciding with foregoing data [51]. When observing long-term heartburn relief, previous studies have reported that up to 93.8% of patients have none or only mild heartburn after LNF [5, 20]. In our study, a full elimination of heartburn was achieved in 83% of the patients, with 95% of the patients expressing satisfaction with its management. Although postoperative PPI use has been relatively common, ranging from 5.8 to 62%, 13% of our patients reported still daily use of PPIs, while additional 4% required only occasional PPI intake [2, 3, 5, 20].

As every chronic disease, GERD has shown to significantly reduce quality of life in patients [4, 8, 52]. Different questionnaires, such as SF-36 and GERD-HRQL, have been used to evaluate potential improvement in quality of life in patients after anti-reflux surgery as a quality measure of the operation itself. It has been shown that quality of life improvement is gradual: achieving symptom relief 1 month after surgery, improvement in GI quality of life in 3 months after surgery, and improvement in general quality of life in the first year after surgery [6, 53]. We found a significant decrease in the GERD-HRQL total score (2 (IQR 0–4.3) vs. 19 (IQR 17–32); *p* < 0.000) after modified LNF, proving a substantial increase in quality of life, confirming previous outcomes. Furthermore, the median alimentary satisfaction given by the patients was 9/10, supporting enduring contentment overall.

Long-term follow-ups have described re-operation rates ranging from 5 to 15% [20]. In our study, a whole of nineteen patients, (*n* = 19, 5%) needed revision surgery due to reoccurrence of GERD-related symptoms, a rate on the low-end of previous literature. Only one case presented as an emergency revision on day 2 postoperatively, due to a paraesophageal hernia recurrence, causing acute dysphagia. Our data demonstrated that modified LNF with a loose 360° wrap does not increase the incidence of GERD recurrence, needing surgical revision.

The follow-up sample size of three hundred and fifty patients (*n* = 350) analyzed in our study is a strong predictor of the validity of our findings. When compared with current literature, a sample size of that quantity is a rare finding, as GERD patients undergone laparoscopic anti-reflux surgery frequently have a good outcome and therefore have no need for further follow-up. Up to date, other than meta-analyses, no study has been published with a long-term LNF follow-up of up to 16 years with a sample size greater than ours [5, 53–58].

What should not be underestimated is the influence of surgical skill, expertise, and experience in the outcomes of surgical management of GERD. Acknowledging the learning curve, technical standardization is a key step in achieving excellent results with low postoperative side-effect rates [11, 59].

Certain limitations of our study, like its retrospective nature should be taken into consideration. Additionally, there was a lack of objective (EFTs) assessment of GERD elimination postoperatively, thus relying purely on subjective patient evaluation of outcomes. However, due to the invasiveness of the procedure and the majority of patients being asymptomatic, we had ethical considerations to indicate the necessity of such a procedure in our large cohort.

## Conclusion

Persistent dysphagia as well as post-Nissen gas-bloat syndrome occurred in 2% and 12.7%, respectively, while satisfactory heartburn relief was achieved in 95% with a revision rate of only 5% after a median follow-up of 4 years. PD should be no reason for avoiding LNF in patients with diagnosed GERD, as PD rates of up to merely 2% occur in specialized centers and LNF not only prevents its adverse effects, but also leads to an increase in quality of life.
